# Botulinum Neurotoxin Type A in paediatric non-neurogenic therapy resistant overactive bladder: a cohort study

**DOI:** 10.1007/s11255-024-04217-z

**Published:** 2024-09-26

**Authors:** Nicklas B. Hougaard, Anders Breinbjerg, Konstantinos Kamperis, Martin Skott

**Affiliations:** 1 Department of Paediatrics and Adolescent Medicine, Aarhus University Hospital, Palle Juul-Jensens, Boulevard 99, 8200 Aarhus N, Denmark; 2 Department of Clinical Medicine, Aarhus University, Palle Juul-Jensens Boulevard 11, 8200 Aarhus N, Denmark; 3 Department of Urology, Aarhus University Hospital, Palle Juul-Jensens, Boulevard 99, 8200 Aarhus N, Denmark

**Keywords:** Botulinum neurotoxin type A, Overactive bladder, Incontinence, Daytime urinary incontinence

## Abstract

**Introduction and objective:**

Intradetrusor Botulinum Neurotoxin Type A (BoNT-A) is an increasingly applied treatment modality for overactive bladder (OAB) in children with refractory urinary incontinence. Despite that, evidence is sparse, and the potential not fully understood. The aim of this study was to evaluate the effectiveness and safety of intradetrusor injection in children with refractory functional OAB and urinary incontinence. Furthermore, we aimed to identify predictors of efficacy and side effects to BoNT-A treatment.

**Materials and methods:**

We conducted a cohort study of children with OAB and urinary incontinence who received intradetrusor injection of BoNT-A in the period 01.01.2016 to 31.12.2020 at our centre. All patients were refractory to standard urotherapy, anticholinergics, mirabegron and the combination of these treatments. Patients with neurogenic bladder were excluded.

Primary endpoint was the reduction on the frequency of urinary incontinence episodes from baseline. Secondary endpoints included urodynamic parameters and uroflowmetry characteristics as well as side effects.

**Results:**

Forty-three children (mean age at first treatment 10.7 ± 1.8, 30 males) were included.

After first treatment, a reduction of ≥ 50% in incontinence episodes was seen in 58% of patients with daytime urinary incontinence (DUI) and 47% of patients with nocturnal enuresis (NE). Adverse events, mainly urinary tract infections (UTI), were reported by 16% of patients after first treatment. Our analysis identified normal cystometric compliance as a significant predictor of treatment effect We estimated the mean duration of effect to be approximately 7 months.

**Conclusions:**

Intradetrusor BoNT-A injection appears to be a safe and effective option in treating refractory urinary incontinent children with overactive bladder. We identified cystometric compliance as a predictor of response. Most children necessitate repeated treatments. Further prospective and controlled studies are necessary in order to fully identify predictors and potential of treatment.

## Introduction

Overactive bladder (OAB), as defined by the International Children’s Continence Society (ICCS) is the presence of urinary urgency with or without urinary incontinence in the absence of urinary tract infection (UTI) or other pathology [1]. In children, OAB is often accompanied by urinary incontinence which is known to impair psychological well-being and social interaction, hence the need for early and effective treatment [2]. Treatment consists of behavioural changes including increased fluid intake, timed voiding, and correct toilet position (standard urotherapy), and subsequently pharmacological treatment if urotherapy alone is inefficient [3]. First-line drug treatment of OAB is anticholinergics, however in refractory cases β3-adrenergic agonists (Mirabegron) or intravesical Botulinum Neurotoxin Type A (BoNT-A) might be used off-label [3].

BoNT-A originates from the bacterium *Clostridium botulinum* and inhibits acetylcholine-driven signal transmission at the neuromuscular junction [4]. When injected into the bladder wall, BoNT-A inhibits the smooth muscle contraction of the detrusor, resulting in a reversible relaxation of the smooth muscle fibres which can be used in treating OAB [4, 5]. Intravesical BoNT-A is regarded relatively safe; however, transient adverse events such as haematuria, dysuria, post-void residuals (PVR), and UTIs have been reported. Less frequently; acute urinary retention with the need of clean intermittent catheterization (CIC) may be seen [4].

Research on intravesical BoNT-A in non-neurogenic children is sparse, even though the treatment was described already in 2006 [6]. Complete resolution of incontinence after treatment has been reported with variable rates ranging from approximately 40–70% [5, 7–10]. Despite, decent response rates reported, taking into consideration that cases are refractory to standard treatment, predictors for response and the optimal timing for repeated treatment are still not fully identified.

In this study, we assessed the effectiveness of BoNT-A injections in managing non-neurogenic OAB expressed by daytime urinary incontinence (DUI) and/or nocturnal enuresis (NE) in children refractory to standard urotherapy, anticholinergics, mirabegron and the combination of these treatments. Furthermore, we identified predictors of treatment response and evaluated the incidence of potential side effects.

## Methods

### Patients and materials

The study was designed as a cohort study performed at Aarhus University Hospital, Aarhus, Denmark, including children (< 18 years of age) that were treated with intravesical BoNT-A from 01.01.2016 to 31.12.2020.

We included urinary daytime incontinent children who received BoNT-A once or repeatedly and had no sign of organic or neurogenic involvement (either evaluated clinically or by a normal magnetic resonance imagining (MRI) scan of columna vertebralis). Information was captured on previous workup, treatment, incontinence episodes, uroflowmetry, cystometric parameters and follow-up data after BoNT-A injections. Cystometric bladder compliance was considered normal at a cut off at > 10 ml/cm H_2_O [11]. Children receiving anticholinergics prior to treatment were asked to withdraw treatment 2–3 weeks after the BoNT-A procedure (as full effect of BoNT-A were expected to have occurred at this time), and children receiving desmopressin were asked to continue this treatment after the procedure.

Prior to treatment all patients were managed and treated according to the guidelines from ICCS [3, 12]. This included standard urotherapy, anticholinergics, mirabegron and the combination of these treatments.

### Intravesical BoNT-A injection procedure

For intravesical injections, BoNT-A (Allergan, Irvine, CA, USA) was diluted in 0.9% saline to 10 international units/ml. Injections were given into the bladder base and dome, with 20–30 injections performed, depending on the total dose, using a transurethral 23-gauge injection needle under rigid cystoscopic guidance (9.8 Fr paediatric cystoscope) at 10 units/kg to a maximum of 300 units. All injections were performed under general anaesthesia with antibiotic prophylaxis given intra-operative as single dose intravenously. Urine was sampled pre-operatively, and if there was clinical suspicion of UTI, the procedure was postponed until any UTI was treated.

### Outcome measures

Primary outcome measure was the number of incontinence episodes per week, in daytime and night-time respectively, before and after intravesical BoNT-A injection at follow-up after approximately 2 months. Following the ICCS criteria, full response was defined as complete resolution of incontinence and partial response was defined as 50–99% reduction in incontinence episodes [1]. Status of incontinence was reported by the parents on follow-up after the procedure.

Secondary outcome measures included adverse events and duration of the effect, evaluated by the time between the primary BoNT-A procedure, and the parent request for re-treatment in children with effect of the treatment.

### Data analysis

Statistical analysis was conducted using R Core Team (R Foundation for Statistical Computing, Vienna, Austria). Baseline data are reported as either categorical or continuous as appropriate. Continuous data are presented as mean with standard deviation (SD). Logistic regression was performed to evaluate predictive factors and risk factors for treatment effect and side effects.

In risk factor analysis the data were dichotomized. Effect was defined as a reduction of symptoms < 50%, and no effect as reduction of incontinence episodes < 50% compared to baseline. Relevant independent clinical outcomes were grouped into dichotomous variables, to enter the logistic regression.

Bladder-size variables, mean voided volume (MVV) and cytometric filling bladder capacity were standardised with expected bladder capacity for age (EBC), using the formula EBC = (30 ml*age in years) + 30 ml, according to ICCS terminology [1].

## Results

### Participants

A total of 43 children with treatment refractory urinary incontinence were eligible for analysis. Baseline data can be seen in Table 1. The cohort consisted of 30 boys, and the mean age in the cohort was 10.7 (SD = 1.8) years. All participants had been treated with urotherapy and anticholinergics with or without the addition of mirabegron. A varying number of children had tried other therapies (Table 1), but without sufficient effect. All participants were refractory to earlier treatments and were thus offered BoNT-A injections.

**Table 1:** Demographics and baseline data

*Demographics* (*n* = 43)	
Sex (male)	30 (70%)
Age (years)	10.72 (1.79)
Overweight( ≥ 1SD)	6
Underweight( ≤1SD)	8
History of UTIs	9 (21%)
*Urinary incontinence* (*n* = 43)	
DUI only	9 (21%)
Combined DUI & EN	34 (79%)
*Severity of DUI*	
Never	1 (2%)
Once a week or less	3 (7%)
2–3 times a week	3 (7%)
Once a day or more	36 (84%)
*Severity of NE*	
Never	9 (21%)
Once a week or less	4 (9%)
Several times a week	5 (12%)
Every night	25 (57%)
*Prior/current treatment* (*n* = 44)	
Urotherapy	43 (100%)
Medical treatment	43 (100%)
Pad and bell alarm	11 (26%)
TENS	23 (53%)
AB prophylaxis	4 (9%)
CIC	1 (2%)
Biofeedback	4 (9%)
*Uroflowmetry* (*n* = 40)	
Voided volume (% of EBC for age)	0.42 (0.21)
Residual urine (≥ 20 ml)	17 (43%)
Max flowrate (ml/s)	21.0 (10.7)
*Shape of curve*	
Bell	20 (49%)
Tower	9 (23%)
Plateau	5 (12%)
Staccato	4 (10%)
Interrupted	2 (5%)
*Home recordings* (*n* = 32)	
*Frequency* (*n*)	
2 to 3 voids/day	2 (6%)
4 to 7 voids/day	20 (63%)
8 to 12 voids/day	9 (28%)
≥ 12 voids/day	1 (3%)
MVV (%EBC for age)	58.7 (20.4)
*Cystometry (n = 43)*	
Detrusor overactivity (*n*)	35 (83%)
End filling pressure (cm H_2_O)	36.14 (43.7)
Capacity (ml/EBC for age)	0.45 (0.19)
Compliance, good (> 10 cm H_2_O/ml)	32 (76%)
*Treatments*	
≥ 2	30 (68%)
≥ 3	8 (18%)
≥ 4	1 (2%)

Data presented as *n* (proportion) or mean (SD)

Individuals with DUI (n = 43) were severely affected, with 82% of the children experiencing incontinence episodes every day. In terms of nocturnal enuresis (NE) severity (*n* = 34), 57% were wet every night. About 80% of the children suffered from DUI and NE in combination (*n* = 34).

Prior to BoNT-A injections, all patients underwent uroflowmetry, revealing that 17 patients (43%) presented with PVRs exceeding 20 ml (mean PVR 22.7 ml, range 0–80 ml). The uroflowmetry curves were normal (bell or tower shape) in 74% of the voids, leaving 26% abnormal (plateau, staccato shape, or interrupted stream).

Home recordings were available for 32 patients (74%). The MVV for the group was 58.7% (SD = 20.4) of EBC.

All patients underwent invasive urodynamics prior to BoNT-A injections, confirming detrusor overactivity in the majority of patients (83%). The average cystometric filling capacity was 45% (SD = 19) of EBC. The bladder compliance was normal in 32 (76%) of children.

### Primary outcomes

In the group of children with both DUI and NE (Table 2) nine patients (26%) experienced full response and 11 (32%) partial response when considering DUI. Furthermore, 58% of the group experienced an improvement in symptoms with a reduction of more than 50% in DUI episodes. For children suffering from NE episodes, nine (26%) experienced full response, and seven (21%) partial response, leaving 18 patients (53%) with no response. Hence 46% of the children reported an improvement in symptoms with a > 50% reduction in enuresis episodes.

**Table 2:** Primary outcomes

	DUI (*n* = 43)			NE (*n* = 34)		
	Full response (100%)	Partial response (50–99%)	No response	Full response	Partial response (50–99%)	No response
Both DUI and NE (*n* = 34)	9 (26%)	11 (32%)	14 (41%)	9 (26%)	7 (21%)	18 (53%)
DUI only (*n* = 9)	1 (12%)	4 (44%)	4 (44%)			

Primary outcomes. Response rate on DUI and NE in relation to baseline demographics (both incontinence types present combined or isolated DUI or NE). Data presented as *n* (proportion)

Six patients (17%) experienced a full response in both DUI and NE.

In children with isolated DUI (*n* = 9) only one experienced a full response (12%), and four (44%) a partial response. Four (44%) children reported no response.

Twenty-six families requested re-treatment. Time of effect was calculated from time of first procedure to request for additional treatment. Relapse of either DUI or NE may have been the reason for re-treatment. The mean time until request for re-treatment was 6.2 months (SD 5.11), with most of the families (58%) experiencing no effect of treatment anymore, and 15 and 27% experiencing < 50% and > 50% of symptoms of pre-treatment baseline, respectively.

### Secondary outcomes

After treatment, seven children (16%) reported side effects. Four children developed an UTI, one reported macroscopic haematuria for more than 3 days, one bladder pain and one had considerably increased post-treatment PVR, however without the need for intermittent catheterization.

Using multivariate logistic regression (Table 3), we were able to identify a normal pre-treatment cystometric compliance (> 10 ml/cm H2O) as a significant (*p* < 0.05) predictor of effect of treatment in DUI.

**Table 3:** Logistic regression for predictive factors for effect. Responders (50–100% reduction in symptoms compared to baseline) vs. non-responders

	DUI (*n* = 43)				NE (*n* = 34)			
	Estimate	SE	*Z* value	*p* value	Estimate	SE	*Z* value	*p* value
Sex (boy vs. girl)	– 1.08	1.29	– 0.84	0.40	0.28	1.43	0.19	0.85
Age^a^	1.58	1.27	1.24	0.21	1.30	1.27	1.02	0.31
Overweight^b^	– 0.59	1.44	– 0.41	0.68	– 0.50	1.64	– 0.31	0.76
Underweight^c^	1.03	1.50	0.68	0.49	1.35	1.45	0.93	0.35
MVV^d^	1.13	1.16	0.98	0.33	2.03	1.56	1.30	0.19
PVR^e^	– 0.88	1.27	– 0.69	0.49	– 3.77	1.97	– 1.91	0.055*
High frequency from FVC^f^	2.82	1.61	1.75	0.08*	2.10	1.55	1.35	0.18
Uroflow curve^g^	2.64	1.73	1.52	0.13	0.15	1.19	0.13	0.90
Cystometric compliance^h^	3.61	1.68	2.15	0.031**	4.8	2.31	2.08	0.037**
Cystometric capacity^i^	3.31	1.87	1.80	0.077*	2.67	1.89	1.41	0.16

^a^Age cut-off, ≥ 11 years as reference

^b^≥1 SD for age as reference

^c^≤ 1 SD for age as reference

^d^Maximum voided volume, standardised as % of EBC, > 50% as reference

^e^Post-void residual prior to treatment, > 20 ml as reference

^f^ > 8 voids per day as reference

^g^Abnormal (disrupted, staccato, plateau) as reference

^h^Cystometric compliance prior to treatment, normal as reference, cut off 10 ml/cm H_2_O

^i^Age standardised with EBC for age, > 50% as reference

*p value  < 0.1

**p value  < 0.05

We found no effect of Body Mass Index (BMI) in the regression. Interestingly, having a PVR higher than 20 ml could in cases with NE be associated, however not significant in our finding (*p* = 0.055).

Regressions for effect were also performed using only full effect individuals as independent variable, but none of these showed significant findings.

We were not able to identify significant risk factors for side effects using multivariate logistic regression (Table 4), but age above 11 years old were suggestive of association with side effects (*p* = 0.066).

**Table 4:** Logistic regression for risk factors of side effects

	(*n* = 43, including 7 with side effects)			
	Estimate	SE	*Z *value	*p* value
Sex	1.40	1.19	1.17	0.24
Age^a^	2.60	1.42	1.83	0.066*
Overweight^b^	0.05	1.48	0.03	0.97
PVR^c^	– 0.48	1.14	– 0.42	0.67
High frequency from FVC^d^	18.14	3100.2	0.006	0.995
Cystometric compliance^e^	– 0.94	1.48	– 0.63	0.52
Cystometric capacity^f^	– 1.33	1.09	– 1.22	0.22

^a^Age cut-off, > 11 as reference

^b^≥1SD for age as reference

^c^Post-void residual prior to treatment, > 20 ml as reference

^d^ > 8 voids per day as reference

^e^Cystometric compliance prior to treatment, normal as reference (cut off 10 ml/cm H_2_O)

^f^Age standardised with EBC for age, > 50% as reference

*p value  < 0.1

**p value < 0.05

## Discussion

This study demonstrates the effect of BoNT-A injections in managing DUI and/or NE in children with non-neurogenic OAB refractory to standard urotherapy, anticholinergics, mirabegron and the combination of these treatments. More than half of the children (58%) experiencing DUI had a reduction in incontinence episodes ≥ 50% following the first treatment. In terms of NE frequency half of the children (47%) had a full or partial response. Six patients with combined DUI and NE experienced a total remission of both their DUI and NE episodes.

Data analysis revealed a significant association between normal pre-treatment cystometric bladder compliance and effect of BoNT-A-injections.

Few studies exist on the efficacy of Botox in children with non-neurogenic OAB. In late 2023 a systematic review was performed by Hoelscher et al*.* [13]. The review included only five relevant cohort studies and meta-analysis could not be performed due to lack and heterogeneity of data. This underlines the current and future need for additional research on the subject.

A recent study by Lambregts et al*.* [8] report a short-time success rate (more than 50% reduction in incontinence episodes) of 72% and the authors were able to identify male sex and small pre-treatment cystometric bladder capacity as suggestive predictors for improving urge incontinence. In our study, we were unable to find a response-association to sex and bladder capacity.Unfortunately, Lambregts et al. did not perform univariate analyses for cystometric bladder compliance, which were our only significant finding in relation to response.

Regarding safety of treatment, we found 16% of children to experience side effects, all of which were transient. This rate corresponds to earlier reported rates and distribution (UTIs more frequent than e.g. PVR) of side effects ranging from 8 to 33% [7–9]. Our regression analysis showed a suggestive association between age above 11 years and the risk of side effects. To our knowledge regression analysis on side effects has not been performed earlier. These findings might be used to risk stratify children after treatment, and tailor the monitoring. Yet, interpretation must be cautious as our dataset is small and findings might be spurious.

Time of effect (either partial or full response) was defined as time until request for additional treatment. We found a median effect-duration of 6.9 months corresponding with earlier studies [8, 10], however effect of up to 12 months has also been reported [6, 7]. In children with neurogenic bladders effect has been previously described to last for years [14].

Equally to our study, the work by Ringoir et al*.* distinguished between if the child had only DUI or NE, or if both types of incontinence were present. This study included 257 children and were able to provide an even higher rate of complete response, especially in relation to isolated EN (50%) or DUI (46%) [7].

This study has certain limitations, especially related to being a non-intervention cohort study. First, the absence of a control group for the comparison of effect of placebo or of drug therapy (anticholinergics and mirabegron). There was no standardised timing of outpatient clinic follow-up after treatment, which could lead to biases in relation to short- and long-term effects.

We found effect of BoNT-A-injections on incontinence in both DUI and NE. However, the captured response was based on patient’s subjective assessment of response and not substantiated by frequency volume charts or home recordings of incontinence episode frequency.

There is need for additional prospective studies to fully evaluate the effect both in short- and long-term, and to provide predictors of response as well as risk factors for side effects.

## Conclusions

We found BoNT-A intravesical injections a safe and effective treatment option in paediatric OAB otherwise difficult to treat. We identified normal pre-treatment bladder compliance as a predictor of response as well as age above 11 years to be suggestively associated with side effects to treatment.

## Data Availability

The data supporting the findings of this study are available upon request from the corresponding author. Access to the data is subject to approval and may be provided in accordance with applicable privacy and ethical guidelines. Please contact corresponding author for further information.

## Abbreviations

BMI: Body Mass Index
BoNT-A: Botulinum Neurotoxin Type A
CIC: Clean intermittent catheterization
DUI: Daytime urinary incontinence
EBC: Expected bladder capacity
ICCS: International Children’s Continence Society
MRI: Magnetic resonance imaging
MVV: Mean voided volume
NE: Nocturnal enuresis
OAB: Overactive bladder
PVR: Post-void residuals
UTI: Urinary Tract Infection
